# *Uropathogenic specific protein* gene, highly distributed in extraintestinal uropathogenic *Escherichia coli*, encodes a new member of H-N-H nuclease superfamily

**DOI:** 10.1186/1757-4749-5-13

**Published:** 2013-06-10

**Authors:** Myo Thura Zaw, Eiki Yamasaki, Shingo Yamamoto, G Balakrish Nair, Keiko Kawamoto, Hisao Kurazono

**Affiliations:** 1 Division of Food Hygiene, Department of Animal and Food Hygiene, Obihiro University of Agriculture and Veterinary Medicine, Obihiro, Hokkaido, Japan; 2 Department of Urology, Hyogo College of Medicine, Nishinomiya, Hyogo, Japan; 3 Translational Health Science and Technology Institute, Gurgaon, Haryana, India; 4 Section of Food Microbiology and Immunology, Research Center for Animal Hygiene and Food Safety, Obihiro University of Agriculture and Veterinary Medicine, Obihiro, Hokkaido, Japan

**Keywords:** Uropathogenic *Escherichia coli*, Pathogenicity island, Uropathogenic specific protein, Non-specific nuclease, H-N-H superfamily

## Abstract

**Background:**

The uropathogenic specific protein (Usp) and three OrfU proteins (OrfU1, OrfU2 and OrfU3) are encoded in the putative small pathogenicity island which is closely associated with Uropathogenic *Escherichia coli*. Although homology search revealed that Usp and OrfUs have a homology with nuclease-type bacteriocins, which possess H-N-H nuclease motif, and immunity proteins respectively, the molecular activity of these proteins was never investigated. In this study, we try to over-express Usp in *E*. *coli*, purify Usp and characterize its molecular activity.

**Method:**

Recombinant Usp protein was expressed in *E*. *coli* BL21(DE3) cells together with 6× Histidine tagged OrfU1 (OrfU1-His) protein, and purified with affinity chromatography using Ni^2+^ chelating agarose. The nuclease activity of the purified Usp was examined *in vitro* by using plasmid DNA as a substrate. The importance of H-N-H motif in nuclease activity of Usp was examined by site-directed mutagenesis study.

**Results:**

We revealed that pET expression vector encoding Usp alone could not be maintained in *E*. *coli* BL21(DE3), and insertion of the *orfUs* as well as *usp* in the constructed plasmid diminished the toxic effect, suggesting that co-expressed OrfUs masked the activity of Usp. To purify Usp protein, we employed the expression vector encoding untagged Usp together with OrfU1-His. A tight complex formation could be observed between Usp and OrfU1-His, which allowed the purification of Usp in a single chromatographic step: binding of Usp/OrfU1-His complex to Ni^2+^ chelating agarose followed by elution of Usp from the complex with denaturing reagent. The purified free Usp was found to have the nuclease activity, and the activity was constitutively higher than Usp/OrfU1-His complex. H-N-H motif, which is found in various types of nucleases including a subfamily of nuclease-type bacteriocin, had been identified in the C-terminal region of Usp. Site-directed mutagenesis study showed that the H-N-H motif in Usp is indispensable for its nuclease activity.

**Conclusion:**

This is the first evidence of the molecular activity of the new member of H-N-H superfamily and lays the foundation for the biological characterization of Usp and its inhibitor protein, OrfUs.

## Background

Urinary tract infections (UTI) are one of the most common infections in human and therefore an important health problem, resulting in 8.2 million physician visits, 1.7 million emergency department visits and 366,000 hospitalizations with an annual projected cost of more than $3.4 billion during the year 2000 in the United States [1]. Uropathogenic *Escherichia coli* (UPEC) are responsible for 80 - 90% of community-acquired UTIs and 40% of nosocomial UTIs [2,3]. UPEC possesses a diverse array of virulence and fitness factors. Adherence factors such as type 1, P, F fimbriae and Dr family adhesin help the UPEC to attach to uroepithelium and establish infection [4]. The UPEC strains also possess an iron uptake system which enables it to survive under iron limiting host environments [5]. UPEC also produces toxins such as alpha-hemolysin and cytotoxic necrotizing factor 1 which can inflict direct damage on the urinary tract tissues [6,7].

The *uropathogenic specific protein* (*usp*) gene was discovered in the UPEC strain Z42 isolated from a prostatitis patient when looking for homologues of the *Vibrio cholerae zot* gene in UPEC [8]. The *usp* gene was predicted to encode a 346 amino acid protein designated as uropathogenic specific protein (Usp). Located downstream of the *usp* gene were three small open reading frames (designated as *orfU1*, *orfU2* and *orfU3*) putatively encoding 98, 97 and 96 amino acid proteins known as OrfU1, OrfU2, and OrfU3 proteins respectively. Although no function has been assigned to Usp, the *usp* gene was reported to be more frequently associated with UPEC strains than fecal *E*. *coli* isolates, and enhance the infectious potential of *E*. *coli* strains in mouse pyelonephritis model, suggesting that Usp may play a role in UPEC pathogenesis [9].

The possibility of Usp to be a bacteriocin was suggested by A. H. A. Parret and R. De Mot, based on sequence homology analysis [10]. In bacteriocin-producing bacteria, immunity protein which inhibits the killing activity of bacteriocin is co-synthesized to protect the producing cell from suicide. The immunity proteins directly bind to the bacteriocins and form a considerably tight complex, which remain stable even after it is released from producing bacterial cells into the environment. The homology analysis revealed that whereas Usp has a homology with nuclease-type bacteriocins such as S-type pyocin produced in *Pseudomonas aeruginosa* and E group colicins produced in *E*. *coli*, OrfUs have a homology with immunity proteins for these bacteriocins [10]. Most nuclease-type bacteriocins possess three functional domains: receptor recognition domain, translocation domain and nuclease domain, which are respectively responsible for recognition of specific receptor protein on target cell membrane, translocation of the protein into target cell and degradation of chromosomal DNA of target cell [11]. The receptor protein varies for each bacteriocin. For example, colicin E7 and E9 of *E*. *coli* bind to the BtuB on *E*. *coli* membrane, [11] and pyocin S1, S2 and S3 of *P*. *aeruginosa* bind to ferripyoverdine receptor on *P*. *aeruginosa* membrane [12]. These specific recognitions contribute to the creation of narrow and species specific killing spectrum of each bacteriocin.

The H-N-H motif is known as a divalent metal ion binding, nucleic acid cleavage-module consisting of 30 to 40 amino acid residues. This motif could be observed in various types of nucleases represented by nuclease-type bacteriocins [13,14] and intron-encoded homing endonucleases [15]. The C-terminal region of Usp shows the homology to H-N-H motif [10]. In this study, to investigate the molecular activity of Usp, we constructed an *E*. *coli* strain overproducing Usp, developed the purification method for Usp, and examined nuclease activity of the purified protein and importance of H-N-H motif in the activity of Usp. The purification method described in this study provides a useful material for further analysis of molecular and biological activity of Usp.

## Results

### Construction of *E*. *coli* strain overproducing recombinant Usp

**Figure 1:**
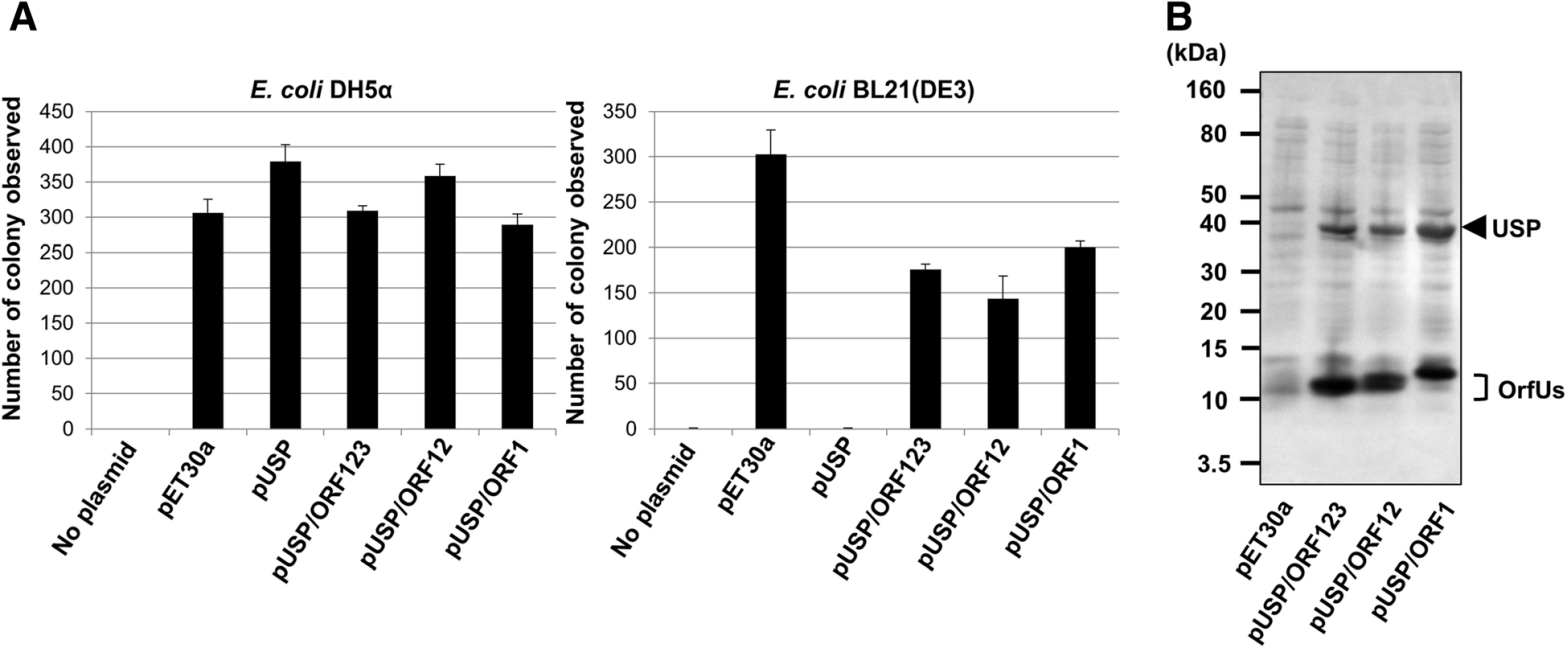
**Construction of the *E***. ***coli *strain overproducing recombinant Usp.** (**A**) Comparison of colony formation by *E. coli* DH5α (left panel) or *E. coli* BL21(DE3) (right panel) after transforming with plasmids encoding Usp alone or Usp together with OrfUs. The plasmids encoding Usp alone or Usp together with OrfUs (for details, see Table 1) were transformed into *E. coli* DH5α or *E*. *coli* BL21(DE3) and selected on kanamycin containing LB agar plate. The number of colony observed on the Φ100 mm plate by each transformed *E*. *coli* after overnight incubation at 37°C is indicated on y-axis. Data are means ± SD of values from three experiments. (**B**) CBB staining analysis of the expression of Usp and OrfUs in transformed *E*. *coli* BL21(DE3). The *E*. *coli* BL21(DE3) cells harboring plasmids indicated were cultured for 3 hour, and then the expression of recombinant proteins was induced by IPTG. Cells were harvested 6 hour post induction. Protein expressions were analyzed by subjecting whole cell protein extracts to SDS-PAGE followed by CBB staining. Molecular weights are estimated based on deduced amino acid sequence of each protein.

In order to construct the *E*. *coli* strain overproducing Usp, the *usp* gene was cloned into T7 based pET-30a vector and the resulting plasmid pUSP was transformed into *E*. *coli* DH5α or *E*. *coli* BL21(DE3) cells. While *E*. *coli* DH5α cells lack a source of T7 RNA polymerase, *E*. *coli* BL21(DE3) cells contain T7 RNA polymerase gene under the control of an isopropyl-β-D-1-thiogalactopyranoside (IPTG)-inducible promoter. Although an abundance of colony formation could be observed when *E*. *coli* DH5α cells were transformed with pUSP and plated on kanamycin containing Luria-Bertani (LB) agar, no colony formation was observed with *E*. *coli* BL21(DE3) transformed with pUSP (Figure 1A). These results were probably due to leaky expression of T7 RNA polymerase in *E*. *coli* BL21(DE3) cells, even in the absence of IPTG, leading to expression of toxic Usp protein. In the case of recombinant protein expression system for known nuclease bacteriocins such as E group colicins, immunity proteins were expressed together with these bacteriocins to protect the host cell from overproduced nuclease bacteriocins [16,17]. As OrfUs are highly homologous with immunity proteins whereas Usp shares homology with bacteriocins [10], we constructed the plasmid containing *usp* gene as well as *orfU* genes. In case of UPEC strain Z42 which was the source of the *usp* gene in this study, there are three *orfU* genes, designated as *orfU1*, *orfU2* and *orfU3*, downstream of *usp* gene. Therefore, we constructed three plasmids as indicated in Table 1 (pUSP/ORF123, pUSP/ORF12 and pUSP/ORF1). The *E*. *coli* BL21(DE3) cells transformed with these plasmids showed significant colony formation on kanamycin containing LB agar (Figure 1A). In addition, significant expression of Usp and OrfUs was observed in these transformed *E*. *coli* BL21(DE3) cells (Figure 1B). These results suggested that OrfU proteins, if expressed together with Usp, could provide the host *E*. *coli* cell with immunity against Usp.

**Table 1:** Plasmids used in this study

**Plasmid names**	**Proteins encoded on the plasmids**
pUSP	Usp-His
pUSP/ORF123	Usp, OrfU1, OrfU2, OrfU3-His
pUSP/ORF12	Usp, OrfU1, OrfU2-His
pUSP/ORF1	Usp, OrfU1-His
pUSP(H314A/H315A)/ORF1	H314A/H315A mutant of Usp, OrfU1-His
pUSP(N330A)/ORF1	N330A mutant of Usp, OrfU1-His
pUSP(H339A)/ORF1	H339A mutant of Usp, OrfU1-His
pUSP(H314A/H315A)	H314A/H315A mutant of Usp-His
pUSP(N330A)	N330A mutant of Usp-His
pUSP(H339A)	H339A mutant of Usp-His

Proteins fused to C-terminal 6× Histidine tag are indicated with “-His”.

### Purification of recombinant free Usp

**Figure 2:**
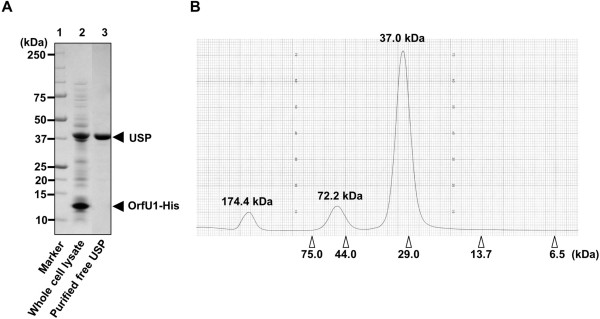
**Purification and molecular weight analysis of free recombinant Usp.** (**A**) Purity analysis of purified free recombinant Usp. Free Usp was purified from *E*. *coli* BL21(DE3) over-expressing Usp and OrfU1-His as described under Methods. Lane 1, marker; lane 2, total cell proteins extracted from *E*. *coli* BL21(DE3) cells over-expressing Usp and OrfU1-His and lane 3, free Usp eluted with 6 M guanidine HCl. (**B**) Elution profile of purified free recombinant Usp from gel filtration column. Free Usp which was dialyzed against 50 mM sodium phosphate buffer was separated by gel filtration column. Opened triangles indicate elution positions of molecular weight markers. Calculated molecular weight was indicated on each peak.

It is generally known that interactions between bacteriocins and its immunity proteins are very strong. Therefore, in the purification of these bacteriocins, bacteriocin/immunity protein complex was first isolated, and separation of the bacteriocins from immunity proteins was done under denaturing conditions or high salt concentrations [16-18]. In this study, we tried to purify the recombinant Usp with a method similar to the purification for nuclease-type bacteriocin, colicin E9 because Usp and OrfU share a high homology with colicin E9 and its immunity protein, respectively [10,16]. To simplify the purification system, we chose the *E*. *coli* BL21(DE3) cells which were transformed with pUSP/ORF1 as a starting material, because Usp could be over-expressed even in the absence of OrfU2 and OrfU3 (Figure 1B). Since C-terminal of OrfU1 protein was fused to 6× Histidine tag, Usp/OrfU1-His complex was first purified with Ni^2+^-chelating agarose and Usp was eluted from Usp/OrfU1-His complex on the Ni^2+^-chelating agarose with the buffer containing 6 M guanidine HCl (Figure 2A). The yield of the purified protein was ~1.3 mg from 1.2 l culture of *E*. *coli* BL21(DE3) cells over-expressing Usp/OrfU1-His complex. After the removal of guanidine HCl, molecular weight of free Usp was examined by gel filtration. Most of the purified free Usp gave the peak corresponding to molecular mass of 37.0 kDa (Figure 2B), which match with the molecular mass of deduced polypeptide of Usp, indicating that free Usp exists mainly as a monomer. This feature is the same with the endonuclease domain of colicin E9 [19,20].

### Nuclease activity of recombinant Usp

**Figure 3:**
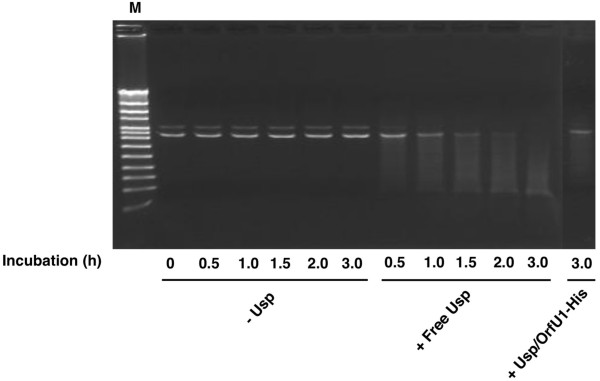
**Nuclease activity of recombinant Usp.** Linear pUC18 (40 ng) was incubated alone or with free Usp (3 μM) or Usp/OrfU1-His complex at 37°C for indicated periods. The concentration of Usp in Usp/OrfU1-His was 3 μM. After incubation, DNA degradation was checked by electrophoresis in 1.0% (w/v) agarose gel. M: Marker.

As the homology analysis revealed that the C-terminal region of Usp showed high homology with nuclease domain of nuclease-type bacteriocins [10], we examined the nuclease activity of purified free Usp. As indicated in Figure 3, the purified free Usp was found to cause non-specific degradation of plasmid DNA in time dependent manner. This result indicated that Usp is a non-specific nuclease similar to other nuclease-type bacteriocins [16]. In addition, we revealed that the nuclease activity of purified Usp/OrfU1-His complex was significantly lower than that of free Usp. These results were consistent with the results observed in Figure 1A.

### Significance of H-N-H motif in nuclease activity of Usp

**Figure 4:**
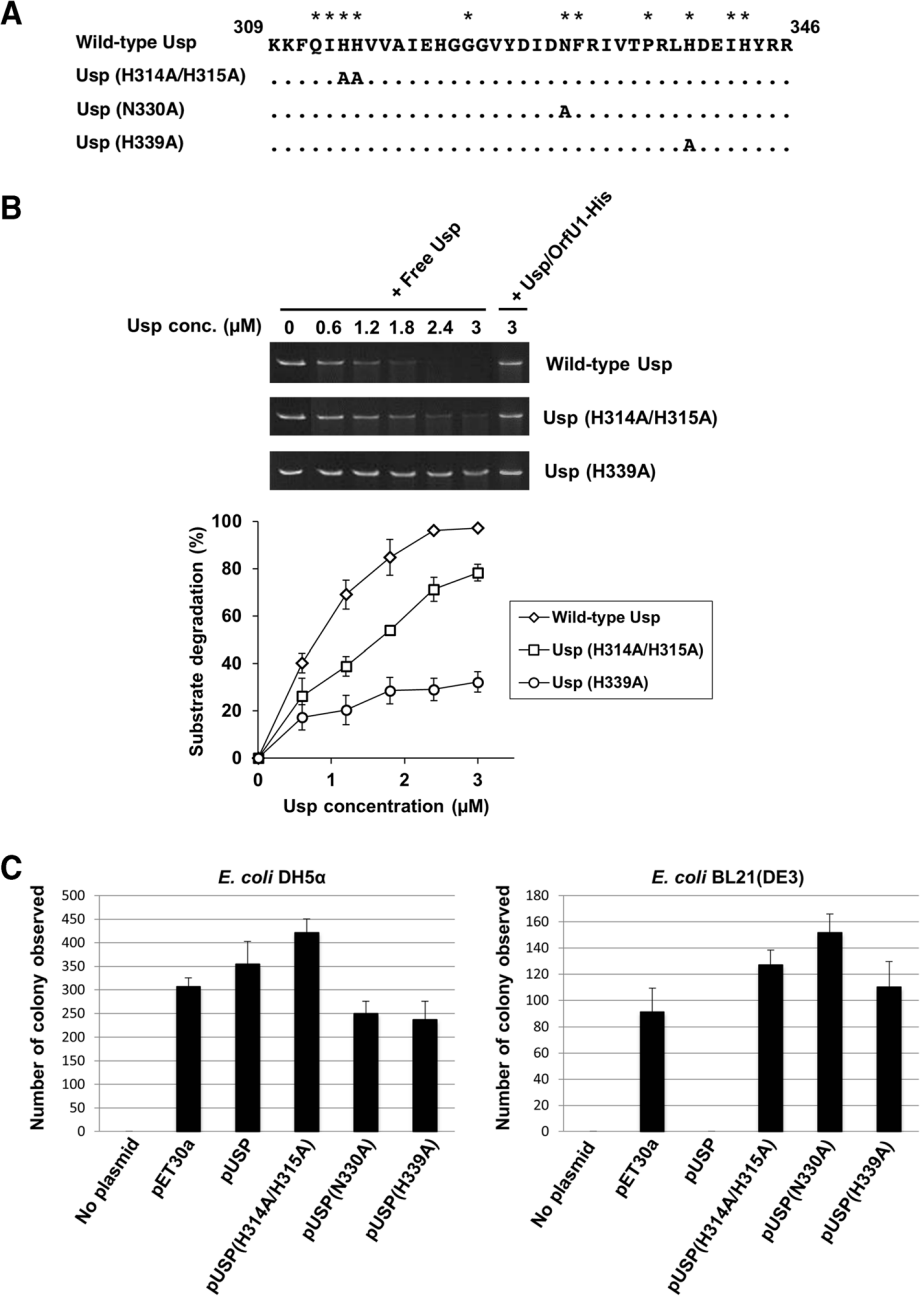
**Significance of H**-**N**-**H motif in nuclease activity of Usp.** (**A**) Sequence alignment of H-N-H motif domain of wild-type and mutant Usps (amino acid position from 309 to 346). Stars indicate the consensus residues in H-N-H motif [14]. Residues without substitution were indicated with dots. (**B**) Nuclease activity assay for mutant Usps. Linear pUC 18 (40 ng) was incubated with indicated concentrations of free Usps or Usp/OrfU1-His complexes. The indicated concentration for Usp/OrfU1-His was the concentration of WT or mutant Usps in each complex. After 2 h incubation, DNA degradation was checked by electrophoresis in 1.0% (w/v) agarose gel. (Upper panel). Quantitative analysis was done by measuring the intensity of each band (Lower panel). Data are means ± SD of values from three experiments. (**C**) Colony formation by *E*. *coli* DH5α (left panel) or *E*. *coli* BL21(DE3) (right panel) after transformation with plasmids encoding mutant Usp alone. The plasmids encoding wild-type or mutant Usps alone were transformed into *E*. *coli* DH5α or *E*. *coli* BL21(DE3) and selected on kanamycin containing LB agar plate. The number of colony observed on the Φ100 mm plate by each transformed *E*. *coli* after overnight incubation at 37°C is indicated on y-axis. Data are means ± SD of values from three experiments.

Multiple sequence alignment revealed that the H-N-H motif is placed in the C-terminal region of Usp [10]. It had been reported that alanine substitutions at the conserved amino acids of the H-N-H motif in colicin E9 attenuate its nuclease activity [14]. In order to investigate whether H-N-H motif is important for nuclease activity of Usp, we employed site-directed mutagenesis studies. In this study, we selected Histidine at position 314, 315 and 339, and Asparagine at position 330 as the mutation sites (Figure 4A). In the mutagenesis, we used pUSP/ORF1 as a template plasmid and the mutant Usps were purified with the same procedure described for wild-type Usp. Although the yields of H314A/H315A mutant and H339A mutant was almost same with that of wild-type Usp, the yield of N330A mutant was very low because the solubility of the mutant protein was low (data not shown). Therefore only H314A/H315A mutant and H339A mutant were used for nuclease activity analysis. As shown in Figure 4B, significant reductions in nuclease activity were observed in both mutants. In addition, we constructed the plasmid encoding mutant Usp alone by site-directed mutagenesis of pUSP (Table 1), and examined the colony formation by the *E*. *coli* BL21(DE3) cells harboring these plasmids. The *E*. *coli* BL21(DE3) cells which were transformed with plasmid encoding H-N-H motif mutant alone gave abundant colony formation (Figure 4C). Taken together, these results suggested that H-N-H is essential motif for the nuclease activity of Usp.

## Discussion

Although *usp* gene could also be detected in non-UPEC isolates, *usp*-positive strains are predominant in UTI isolates [21,22]. In addition, based on its ability to confer infectious potential to non-pathogenic *E*. *coli*, Usp is thought to be an important factor responsible for UPEC infection [9]. But, the mode of action or molecular activity of Usp has never been investigated, because purification method for Usp protein was not established. In this study, we constructed recombinant *E*. *coli* strain in which Usp was over-expressed. Based on its homology with nuclease-type bacteriocins [10], Usp is thought to be a nuclease protein. The over-expression of nuclease proteins, either of eukaryotic or prokaryotic origin, in *E*. *coli* cells is problematic due to cellular toxicity of these proteins. It is impossible to over-express these proteins in *E*. *coli* without employing certain strategies to reduce their toxicity, and co-expression with inhibitor proteins is one of the frequently used strategies [16-18,23,24]. In this study, we chose OrfUs as the inhibitor protein for Usp because OrfUs have a high homology with immunity proteins for nuclease-type bacteriocins [10], and the co-expression method successfully reduced the cellular toxicity of Usp. Three types of *orfUs* (*orfU1*, *orfU2* and *orfU3*) had been identified in the downstream region of *usp*[25]. Moreover, *usp* had been divided into two subtypes based on its sequence, designated as *uspI* and *uspII*. The putative pathogenicity islands consisting of *usp* and *orfUs* (PAIusp) contains one *usp* and two or three *orfUs*, and have been classified into four subtypes according to their sequential patterns [21,22,25]. Comparison of these PAIusp revealed that *uspI* is closely linked with immediately downstream *orfU1* whilst *uspII* is linked to *orfU2*. On the basis of this observation, it had been speculated that OrfU1 is responsible for immunity against UspI whereas OrfU2 is responsible for UspII [10]. The *usp* gene used in this study was cloned from the UPEC strain possessing *uspI* gene. The results obtained in this study indicate that OrfU1 could provide immunity against UspI in the absence of OrfU2 and 3. Although we have not examined capability of OrfU2 and 3 as an immunity protein, we have revealed that OrfU2 could not bind to UspI in the presence of OrfU1 (data not shown).

The H-N-H motif, the importance of which in the nuclease activity of Usp has been revealed in this study, can be found in numerous nucleases other than bacteriocins. The most well known member of this H-N-H nuclease superfamily is the homing endonuclease, which catalyze intron and intein mobility. Moreover, the H-N-H motif is found in a range of nucleases which participate in various biological processes including recombination, programmed DNA rearrangement during differentiation, and phage packing [26]. The motif can also be observed in the active center of some restriction enzymes [27]. These nucleases are produced in wide-range of hosts including bacteria, viruses and eukaryotes. Although several of these nucleases are not well-characterized, all the characterized nucleases with H-N-H motif other than bacteriocins are site-specific endonucleases whereas bacteriocins are non-specific endonucleases. In this study, we revealed that Usp is a non-specific nuclease similar to bacteriocins. Sequence homology between Usp and nuclease-type bacteriocins, including colicins produced by *E*. *coli*, have been previously suggested [10]. Generally, nuclease-type bacteriocins can be divided into three domains: a nuclease domain, a translocation domain and a receptor recognition domain. Sequence alignment revealed that, in addition to the nuclease domain in which H-N-H motif was conserved, Usp has a region homologous to the translocation domain. However, Usp lacks the region homologous to the receptor recognition domain. A receptor recognition domain of bacteriocins is required for binding to specific membrane receptor on target bacterial cells. It is considered that these specific interactions between receptor recognition domains and membrane receptors regulate the narrow killing spectrum of bacteriocins. It is known that the narrow killing spectrum of bacteriocins is usually restricted to the strains of the same or closely related species [28,29]. Although we demonstrated that Usp has a non-specific nuclease activity similar to known nuclease-type bacteriocins including colicins, we had not yet found susceptible *E*. *coli* strain which was killed by purified free Usp or Usp/OrfU1 complex (data not shown). Based on the sequence homology, it has been assumed that Usp and colicins, both of them produced in *E*. *coli*, share the bacteriocin activity, but the killing spectrum of these proteins might differ considerably.

Within the nuclease-type bacteriocins, the difference in ability to degrade RNA was reported. Colicin E9 was reported to cleave short ssRNA whereas colicin E2 did not have RNase activity against phage RNA [20,30], although both of them belong to H-N-H nuclease superfamily. Therefore, we examined RNase activity of free Usp, but we could not detect any RNase activity against short ssRNA at least under the same condition as DNase activity assay (Additional file 1: Figure S1).

## Conclusion

In this study, we could successfully establish a purification method for recombinant Usp which possesses non-specific nuclease activity. The H-N-H motif conserved in the C-terminal region of the Usp was indispensable for its nuclease activity, indicating Usp is the new family member of H-N-H nuclease superfamily. Although Usp is considered as an important virulence factor of UPEC infection based on the previous result of mouse UTI model [9], the role of Usp in UTI has not been investigated. In addition, there is a possibility that Usp also participates in infections outside of urinary tract because *usp* can detected in some non-uropathogenic *E*. *coli* isolates. The purified protein obtained in this study would facilitate an analysis of biological activity and role of Usp during *E*. *coli* infections.

## Methods

### Bacterial strains

*ECOS* competent *E*. *coli* DH5α cells (NIPPON GENE CO., LTD.) were used for construction and maintenance of recombinant plasmids. *ECOS* competent *E*. *coli* BL21(DE3) cells (NIPPON GENE CO., LTD.) were used for expression of recombinant proteins. UPEC strain Z42 was used as source of *usp* and *orfU* genes [8].

### Construction of the plasmids encoding Usp

The uspF1 PCR primer was designed to introduce *Nde* I site containing start ATG codon of *usp* gene. The uspR1, orfu3R1, orfu2R1, orfu1R1 PCR primers were designed to fuse 3′-end of *usp*, *orfU3*, *orfU2* or *orfU1* genes to 6× Histidine tag coding sequence of pET30a vector (Table 2). For amplification of DNA fragments encoding Usp, Usp + OrfU1 + OrfU2 + OrfU3, Usp + OrfU1 + OrfU2, or Usp + OrfU1, the uspR1, orfu3R1, orfu2R1, or orfu1R1 PCR primers were used as reverse primers respectively, whereas the uspF1 primer was used as a forward primer for all clones. The PCR was run by using these primers and the whole cell DNA of UPEC strain Z42 as a template. The amplification reaction was performed by using PrimeSTAR® Max DNA Polymerase kit (Takara Bio Inc.) with 30 cycles of optimized condition (98°C, 10 sec.; 55°C, 15 sec.; 72°C, 60 sec.). The size of amplified PCR fragment was analyzed by agarose gel electrophoresis by using DNA Molecular Marker XVI (Roche Diagnostics) as a molecular weight marker. After TOPO cloning was done for the amplified DNA fragments by using TOPO TA Cloning Kit for Sequencing (Life Technologies Co.), DNA fragment encoding Usp and OrfUs were extracted with *Nde* I and *Xho* I, and cloned into the multiple cloning site of pET30a expression vector by the same restriction enzyme site. The *ECOS* competent *E*. *coli* DH5α cells (NIPPON GENE CO., LTD.) were used as a cloning host, and plasmid purification was done with QIAprep Spin Miniprep kit (QIAGEN).

**Table 2:** Primers used in this study

**Primers**	**Sequence**
uspF1	5′-aacatatgctactgttcccgagtag-3′
uspR1	5′-aactcgagtctcctgtagtgaatttc-3′
orfu3R1	5′-aactcgagttctgaatctttgaacaaag-3′
orfu2R1	5′-aactcgagtctactttgtttagagtctttaaac-3′
orfu1R1	5′-aactcgagtttagagtctttaaacaaggg-3′
HH314AAF	5′-gttaagaaattccagata**gc**t**gc**tgtagttgctatagaacatgg-3′
HH314AAR	5′-ccatgttctatagcaactaca**gc**a**gc**tatctggaatttcttaac-3′
N330AF	5′-gtggagtgtatgatattgat**gc**ttttaggattgttacgcccc-3′
N330AR	5′-ggggcgtaacaatcctaaaa**gc**atcaatatcatacactccac-3′
H339AF	5′-gattgttacgccccgacta**gc**tgatgaaattcactacagg-3′
H339AR	5′-cctgtagtgaatttcatca**gc**tagtcggggcgtaacaatc-3′

Restriction enzyme sites are underlined and sites of mutations in mutagenic primers are underlined and bold.

### Purification of Usp/OrfU1-His complex and free Usp

The *ECOS* competent *E*. *coli* BL21(DE3) (NIPPON GENE CO., LTD.) was used as the host strain for expression of recombinant Usp/OrfU1-His complex. The transformed *E*. *coli* BL21(DE3) was cultured in LB broth supplemented with kanamycin (25 μg/ml) and glucose (1%) at 30°C overnight with vigorous shaking (pre-cultivation). The pre-culture was inoculated into fresh LB broth supplemented with kanamycin (25 μg/ml) and glucose (1%), and further incubation was done at 30°C for 3 h before induction of recombinant protein expression by addition of IPTG (0.1 mM). Six hour after induction, the bacterial cells were harvested by centrifugation, resuspended in the binding buffer (20 mM Tris, 0.5 M NaCl, 5 mM imidazole, pH 7.5), and disrupted by sonication. The cleared cell lysate obtained after the centrifugation of the cell sonicate at 18,000×*g* for 30 minutes was applied onto the column filled with Ni-NTA agarose (QIAGEN) which was equilibrated with the binding buffer. The column was sequentially washed with the binding buffer, the washing buffer (20 mM Tris, 0.5 M NaCl, 60 mM imidazole, pH 7.5), and the binding buffer again. In the case of Usp/OrfU1-His complex purification, the complex was eluted by the elution buffer (20 mM Tris, 0.5 M NaCl, 250 mM imidazole, pH 7.5). On the other hand, the free Usp was eluted with the Guanidine-containing buffer (20 mM Tris, 0.5 M NaCl, 6 M guanidine-HCl, pH 7.5). The eluted fraction was further incubated with Ni-NTA agarose for 1 h with rotation to remove residual OrfU1-His. Obtained Usp/OrfU1-His complex or free Usp was dialyzed against a potassium phosphate buffer (0.02 M KH_2_PO_4_, 0.03 M K_2_HPO_4_, pH 7.0) to remove excess salts or guanidine HCl before performing the nuclease activity assay. The over-expressed proteins and purified proteins were analyzed by MALDI-TOF MS with the view to confirm the identity of each protein by database search. The MALDI-TOF/TOF MS was performed on an AutoFlex II TOF/TOF mass spectrometer (Bruker Daltonics) in accordance with the manufacturer’s instructions. The data acquisition, processing and database search was performed on instrument-specific software: FlexControl, FlexAnalysis, BioTools connected to Masccot search engine with NCBI nr database.

### Determination of protein concentration

Concentrations of purified free Usps were determined by Bio-Rad Protein Assay Dye Reagent Concentrate (Bio-Rad) by using bovine serum albumin as a standard. For concentration measurement of Usp in purified Usp/OrfU1-His complex, purified Usp/OrfU1-His complex was separated with sodium dodecyl sulphate-polyacrylamide gel electrophoresis (SDS-PAGE) followed by Coomassie Brilliant Blue (CBB) staining, and the band intensities of Usp was determined. And the concentration of Usp in Usp/OrfU1-His complex was calculated from standard curve representing the relationship between free Usp concentration and band intensities. Determination of band intensities was performed by image processing and analysis software ImageJ (http://imagej.nih.gov/ij/).

### Analytical gel filtration

Gel filtration chromatography was used to determine the molecular weight of free Usp. The purified free Usp and 5 standard proteins from Gel Filtration LMW Calibration Kit (GE Healthcare Life Sciences) were dialyzed against sodium phosphate buffer (0.05 M NaH_2_PO_4_, 0.05 M Na_2_HPO_4_, 0.2 M NaCl pH 8.0), and separated on the Superdex 75 10/300 GL Column (GE Healthcare Life Sciences) equilibrated with the same buffer. The calibration curve was drawn by plotting of elution volume and LogM_r_ of 5 standard proteins, and molecular weight of each peak obtained from purified free Usp sample was determined from the curve.

### Construction of Usp mutants

Site-directed mutagenesis was carried out by the methods described in QuickChange Lightening site-directed mutagenesis kit (Agilent Technologies). Purified pUSP/ORF1 or pUSP plasmids were used as the template. Mutagenic primers used were HH314AAF and HH314AAR for H314A/H315A mutant; N330AF and N330AR for N330A mutant and H339AF and H339AR for H339A mutant. The sequences of these primer are shown in Table 2. The DNA sequence of the products were confirmed by DNA sequencing. The sequence analysis was done at Hokkaido System Science Co., Ltd. with the ABI PRISM 3130 Genetic Analyzer using the BigDye Terminator v3.1 kit (Applied Biosystems). Expression and purification of these mutant proteins were done following the same procedures as wild-type Usp.

### Nuclease activity assay

Linearized pUC18 plasmid, which was digested by *Sal* I, was incubated with wild-type or mutant Usps in reaction buffer (0.05 M Tris, 80 mM NaCl, 10 mM MgSO_4_, pH 8.0) at 37°C. After incubating for desired time, reactions were stopped by addition of 6× Loading Buffer (TaKaRa Bio. Inc.), and products were separated by electrophoresis through 1.0% (w/v) agarose gel and the DNA was visualized by ethidium bromide staining. For quantitative analysis, the extent of degradation was quantified by densitometric measurement of substrate DNA with Image J.

## Abbreviations

UTI: Urinary tract infection; UPEC: Uropathogenic *Escherichia coli*; Usp: Uropathogenic specific protein; LB: Luria-Bertani; IPTG: Isopropyl-β-D-1-thiogalactopyranoside; SDS-PAGE: Sodium dodecyl sulphate-polyacrylamide gel electrophoresis; CBB: Coomassie Brilliant Blue.

## Competing interests

The authors declare that they have no competing interests.

## Authors’ contributions

MTZ constructed the purification method and carried out the nuclease activity analysis, and drafted the manuscript. EY designed the study and constructed the purification method. SY and HK conceived the study and provide *E*. *coli* strains, and co-drafted the manuscript. GBN and KK participated in the design of the study and co-drafted the manuscript. All authors have approved the manuscript.

## Supplementary Material

Additional file 1: Figure S1 RNase activity of recombinant Usp. The 10 mer ssRNA (GUC GUA AGA G) (60 μg/ml) or 20 mer ssRNA (GUC GUA AGA GGU CGU AAG AG) (20 μg/ml) was incubated alone (Negative Control) or with free Usp (3 μM), Usp/OrfU1-His complex, or RNaseA (7.5 μg/ml) (Roche Diagnostics) in reaction buffer (0.05 M Tris, 80 mM NaCl, 10 mM MgSO_4_, pH 8.0) at 37°C for 3 h. The concentration of Usp in Usp/OrfU1-His complex was 3 μM. After incubation, reactions were stopped by addition of RNA Loading Buffer (TaKaRa Bio. Inc.). The RNA degradation was checked by electrophoresis in 18% (w/v) acrylamide: bisacrylamide (19:1) / 7 M urea / 0.5xTBE gel followed by visualization with ethidium bromide. The 14–30 ssRNA ladder marker (TaKaRa Bio. Inc.) was used as a molecular size marker (M). Click here for file
